# Individual variation in reaction norms but no directional selection in reproductive plasticity of a wild passerine population

**DOI:** 10.1002/ece3.8582

**Published:** 2022-02-14

**Authors:** Heung Ying Janet Chik, Catalina Estrada, Yiqing Wang, Priyesha Tank, Alex Lord, Julia Schroeder

**Affiliations:** ^1^ 4615 Department of Life Sciences Imperial College London Ascot UK

**Keywords:** avian reproduction, climate change, individual variation, natural selection, Phenotypic plasticity, reaction norm

## Abstract

In the plant*–*insect*–*insectivorous bird food chain, directional changes in climate can result in mismatched phenology, potentially affecting selection pressures. Phenotypic plasticity in the timing of breeding, characterized by reaction norm slopes, can help maximize fitness when faced with earlier prey emergence. In temperate passerines, the timing of tree budburst influences food availability for chicks through caterpillar phenology and the resulting food abundance patterns. Thus, the timing of tree budburst might serve as a more direct proxy for the cue to time egg‐laying. The evolutionary potential of breeding plasticity relies on heritable variation, which is based upon individual variation, yet studies on individual variation in plasticity are few. Here, we tested for the laying date—budburst date and the clutch size—laying date reaction norms, and examined 1) the among‐individual variance in reaction norm intercepts and slopes; and 2) the selection differentials and gradients on these intercepts and slopes. Using long‐term data of oak (genus Quercus) budburst and blue tit (*Cyanistes caeruleus*) reproduction, we applied within‐subject centering to detect reaction norms, followed by bivariate random regression to quantify among‐individual variance in reaction norm properties and their covariance with fitness. Individuals significantly differed in intercepts and slopes of both laying date—budburst date and clutch size—laying date reaction norms, and directional selection was present for an earlier laying date and a larger clutch size (intercepts), but not on plasticity (slopes). We found that individuals have their own regimes for adjusting egg‐laying and clutch size. This study provides further support of individual variation of phenotypic plasticity in birds.

## Graphical abstract

In the plant—insect—insectivorous bird food chain, directional changes in climate can result in mismatched phenology, potentially affecting selection pressures. Here we found that individual blue tits display variation in egg‐laying and clutch size plasticity, and that selection has pushed for an earlier laying date and a larger clutch size but does not act on plasticity. This study provides further support of individual variation of phenotypic plasticity in birds.

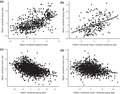

## 1 INTRODUCTION

Climate change poses many impacts on ecosystems, one of which being phenological mismatch, or the mistiming of life‐history events in different trophic levels of a food chain. In a food chain, the peak in abundance of a food source often temporally coincides with that of the food demand of higher trophic levels, as such synchronizing phenological events across trophic levels. However, as temperatures continue to rise (IPCC, 2018), spring phenological events in over 1700 species have advanced at an average rate of 2.3 days per decade (Parmesan & Yohe, 2003). In particular, the plant–insect–insectivore system, namely tree budburst (Badeck et al., 2004; Menzel et al., 2001), insect emergence (Roy & Sparks, 2000), and avian breeding (Both et al., 2005) have experienced phenological advancement.

**FIGURE 1:**
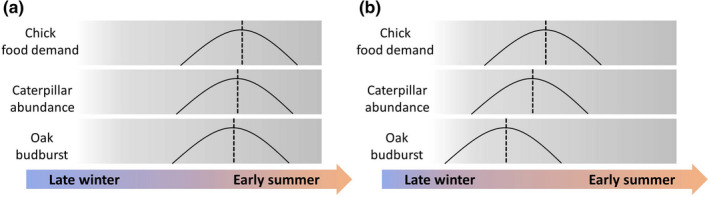
Schematic diagram of phenological shifts. (a) depicts phenological match where oak budburst, caterpillar emergence, and chick food demand align with one another. Note a small time lag still exists. (b) depicts increased mismatch under advancing spring, where laying date of birds remains late, and chick food demand peaks later than the peak of food availability

Phenological advancements can lead to a mismatch when the dependent predator and prey phenologies undergo temporal shifts of different magnitudes, variance, or directions. These mismatches occur when response mechanisms to changing environments differ among species. For example, leaf development and leaf palatability to folivorous insects are largely dependent on temperature in oaks (genus *Quercus*; Buse et al., 1999). In folivorous insects such as the winter moth (*Operophtera brumata*), egg‐hatching and caterpillar emergence is directly dependent on accumulated heat (Dewar & Watt, 1992; Embree, 1970) as well as the number of frost days (Visser & Holleman, 2001). In contrast, insectivorous birds such as great tits (*Parus major*) and blue tits (*Cyanistes caeruleus*) have a more complex response mechanism. In these birds, selection is strongest when chicks are rapidly growing and have the greatest need for high quality food, typically caterpillars (Charmantier et al., 2008). As there exists a time lag of several weeks between the start of breeding and egg‐hatching, females must use environmental cues at the time of egg‐laying to predict the conditions and food availability in the future, and time their egg‐laying accordingly. These cues can correlate but not necessarily causally relate to food availability, and as a result of climate change, might become unreliable (Bonamour et al., 2019). Such unreliable cues can be explained two‐fold (Visser et al., 2004)—(1) climate change affects the environment at the time of decision‐making differently than it affects the environment at the time of selection; or (2) birds may rely on different and/or multiple cues to different degrees, some of which would remain relatively stable (e.g., photoperiod). Either case can lead to phenological mismatch (Figure 1).

In insectivorous birds, such mismatch can result in increased selection pressure for earlier laying dates (Both & Visser, 2001; van Noordwijk et al., 1995; Visser & Gienapp, 2019; Visser et al., 1998). While this selection on laying date has been reported to induce a microevolutionary response at the population level (Gienapp et al., 2008; Husby et al., 2011), evidence for it is sparse. Indeed, a microevolutionary response might be less important for birds to adapt to shifting prey phenology, because laying date is only moderately heritable, leading to microevolution being a slow process and unable to keep up with the more rapid prey phenological changes (Charmantier & Gienapp, 2014; Gienapp et al., 2008). Instead, phenotypic plasticity might play a greater role in adaptation to rapidly changing environments (Gienapp et al., 2008).

**FIGURE 2:**
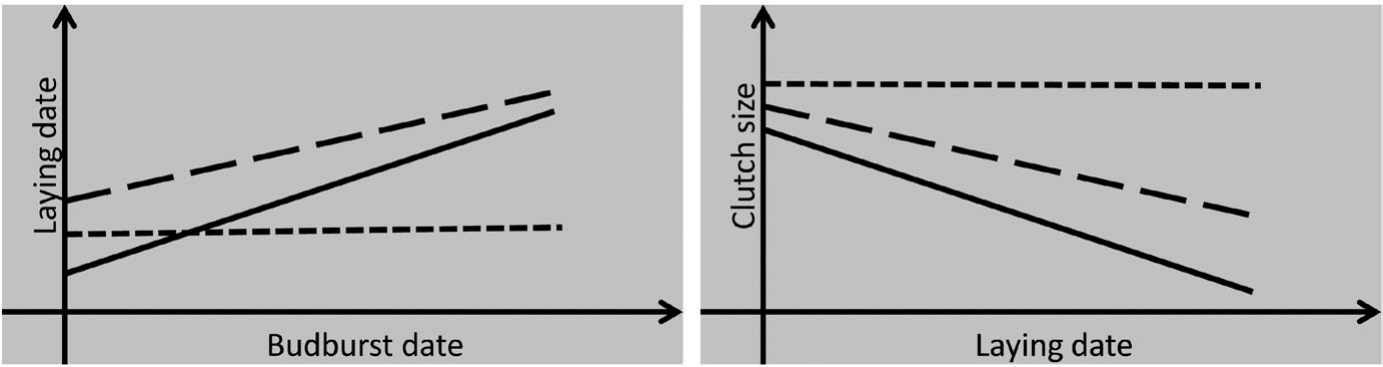
Schematic diagrams of laying date—budburst date and clutch size—laying date reaction norms examined in this study. Lines represent linear regressions of individual reaction norm, which differ in intercept (mean trait value of the individual) and slope (plasticity). Solid line represents a more plastic reaction norm; dashed line represents a less plastic one, and dotted line represents a non‐plastic one. Note the reaction norm intercept can be uncorrelated with the slope (as presented in the laying date—budburst date panel)

Phenotypic plasticity is the expression of more than one phenotypic value from a single genotype or individual across changing environments (Scheiner, 1993). Plasticity can be characterized by the reaction norm, a regression line of phenotypic values over an environmental gradient, where the intercept represents the trait value at the average environment (given the environmental variable is mean‐centered), and the slope represents plasticity (Figure 2, Stearns, 1989). Plasticity can be found in avian life‐history traits such as laying date (Charmantier et al., 2008; Nussey et al., 2005; Porlier et al., 2012; Thorley & Lord, 2015). There is also evidence that plasticity in laying date varies among individuals and is heritable; thus, it could be subjected to evolution under selection pressure (Nussey et al., 2005).

The aforementioned studies on plasticity have largely focused on laying date—temperature reaction norms, using a sliding window approach to identify the climatic window that best predict laying date as the most plausible temperature cue (Nussey et al., 2005; Porlier et al., 2012; Thorley & Lord, 2015). However, the actual cues used by birds to time egg‐laying remain unknown, and literature has suggested other cues such as changes in vegetation (Bourgault et al., 2010; Thomas et al., 2010) and rainfall (Shaw, 2017). There is also growing evidence that cues differ among species and populations (Bonamour et al., 2019; Nilsson & Källander, 2006). While temperature may be the main underlying driver of spring phenology in the plant–insect–insectivore system, it is ultimately tree budburst that constrains the emergence of food peaks of emerging insects, thus forming the basis of synchrony among trophic levels (Dewar & Watt, 1992). Therefore, as budburst date could be a more direct proxy of the underlying cue to time egg‐laying, we explored laying date*—*budburst date reaction norms in this study.

We also considered plasticity in clutch size, as this trait is closely related to fitness (Rowe et al., 1994). The interaction between clutch size and laying date results in a trade‐off to determine an optimal clutch size for every laying date (Lack, 1954). This optimization is governed by two considerations: that reproductive value of an egg declines seasonally with laying date; and that maximum possible clutch size increases with laying date, because reproduction is energetically costly, and food is scarce in the early breeding season (Daan et al., 1990; Drent & Daan, 1980). Overall, these considerations result in clutch size decreasing with laying date (Figure 2, Brommer et al., 2003). A more plastic female may be advantageous and favored over a less plastic one since she can better adjust her optimal clutch size—should she lay early, she could lay more eggs with higher reproductive values; should she lay late, she could better minimize phenological mismatch, since a smaller clutch means she can more quickly proceed to incubation, as most insectivorous passerines are constrained to lay a maximum of one egg per day (Perrins, 1979). Therefore, we are also interested in plasticity as the slope of clutch size—laying date reaction norms.

Despite their importance, little research exists that examines laying date—budburst date and clutch size—laying date reaction norms and their evolution. In this study, we characterized the laying—budburst date and clutch size—laying date reaction norms and their individual variation, as individual variation provides the basis for heritable variation, which allows for microevolution (Falconer & Mackay, 1996). Using long‐term data of a wild blue tit (*Cyanistes caeruleus*) population, we employed within‐subject centering (van de Pol & Wright, 2009) and bivariate random regression models (Arnold et al., 2019) to test for three hypotheses: 1) that the laying date—budburst date and clutch size—laying date reaction norms exist in our population, 2) that there is among‐individual variance in the intercept and slope of the reaction norms, allowing the possibility of an evolutionary response; and 3) that there is selection on these reaction norm properties.

## 2 METHODS

### 2.1 Study species and site

This study used long‐term data of a nest‐box population of blue tits at Silwood Park, UK (51°24'N, 0°38'W). The blue tit is a small passerine that commonly dwells in deciduous or mixed woodlands and breeds readily in holes or nest boxes (Svensson et al., 2011). Its breeding season commences in late March and typically lasts until June, where females lay a single brood with up to 19 eggs according to previous records and feed their young with predominantly caterpillars. The study site has an area of approximately 100 ha and consists of deciduous woodlands of varying ages. The site is dominated primarily by oak trees including the English oak (*Quercus robur*), among other deciduous tree species. Within the site, 200 nest boxes were installed in 2002, with further changes in subsequent years, totaling 259 nest boxes as of 2019.

### 2.2 Data collection

We used blue tit breeding data collected in 2002–2019 (data from 2014 was unavailable). Every year data collection began in late March, and nest boxes were examined every other day for signs of nest‐building and egg‐laying. We recorded the laying date, defined as the date on which the first egg of each clutch is laid, in “April Days,” the number of days passed since the 1st of April (= Day 0) in a given year. Upon allowing 15 days for females to complete their clutches, we caught blue tits in their nest boxes and recorded the final number of eggs laid as the clutch size. Birds were identified by uniquely numbered metal rings from the British Trust for Ornithology (BTO), and sexed by the presence of a brood patch, a patch of featherless, highly vascularized skin on the abdomen of females. We then allowed 11 days before revisiting nests to check for egg hatching, upon which hatching date is recorded. We measured, weighed, and fitted chicks with BTO metal rings and counted the number of ringed chicks. From 2002 to 2011, this was done when chicks were 7 days old, and from 2012 onward, when chicks were 14 days old. We recorded the number of dead chicks and calculated the number of fledglings by revisiting nest boxes when chicks were 19 days old.

**FIGURE A1:**
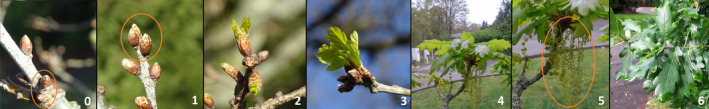
Leaf stages at different scores: 0 = Bud dormant; 1 = Green showing at bud tip; 2 = Bud elongation and budburst; 3 = Leaves unfurling; 4 = Leaves fully extended; 5 = Anther developing; 6 = Leaves tanninized. Photographs by Doblas (2017)

Oak leaf budburst phenology was monitored from 2007 to 2019. We monitored in total 3945 oak trees, each of which was assigned a unique ID and a nest box territory of 25‐m radius around a nest box based on the average breeding territory of blue tits (Arriero et al., 2006). Ten per cent of trees are monitored annually, and the remaining 90% biennially. Each year, we carried out tree monitoring from March to May. We visited each tree every two or three days and recorded the overall budburst score for that tree from not yet budding (stage 0) to fully tanninized (stage 6, Appendix Figure A1) based on the majority of its leaves, until all trees have been scored at stage 6. Dates on which a tree reached a certain stage were recorded as April Days.

### 2.3 Laying date—budburst date and clutch size—laying date relationships

We ran all models in R v.3.5.1 (R Core Team, 2020). Laying date, budburst date, and clutch size data were mean‐centered per the definition of a reaction norm. After data exploration and visual inspection, we approximated both laying date and clutch size with a Gaussian distribution. Using a Poisson distribution for clutch size drew the same conclusions (data not shown). For each breeding record, we calculated the average date on which the oak trees corresponding to their paired nest box reached stage 2, when the bud is elongated and leaves first emerge. At this stage, caterpillars first start feeding on the leaves (van Dongen et al., 1997), making this stage most representative of the cue for bird egg‐laying. To examine the relationships between the traits, we used the R package *lme4* (Bates et al., 2015) to run two linear mixed models, one with laying date as the response and budburst as the explanatory variable, and another with clutch size as the response variable and laying date as the explanatory variable. In both models, we fitted bird ID and year as random effects on the intercept to account for repeated measures of the same individuals and in the same year respectively.

### 2.4 Individual laying date—budburst date and clutch size—laying date reaction norms

To examine whether individual reaction norms and plasticity exist in these traits, we employed within‐subject centering (van de Pol & Wright, 2009), which allows apparent laying date—budburst date and clutch size—laying date relationships to be differentiated into within‐individual effects (individuals displaying different laying dates at different budburst dates, and different clutch sizes at different laying dates) and between‐individual effects (early‐laying birds always sampled in early budburst years, and birds with larger clutch size always laying early), the former of which signifies individual plasticity. After excluding one‐time breeders, we mean‐centered budburst date and laying date for each individual bird so that observations represented deviations from the individual mean, essentially eliminating between‐individual variation in budburst date. With these new values, we built two linear mixed models, one with within‐individual mean‐centered budburst date as the explanatory variable and laying date as the response, and another with within‐individual mean‐centered laying date as the explanatory variable and clutch size as the response. We fitted bird ID as a random effect in both models. The model formula for the within‐individual relationship between budburst date and laying date is presented below as an example.

$$\text{LD}∼\left(\text{BD}‐{\bar{\text{BD}}}_{\text{ID}}\right)+\left(1|\text{ID}\right)$$

where LD is the laying date, BD is the budburst date, and ${\bar{\mathrm{BD}}}_{\mathrm{ID}}$ is the average budburst date observed for each individual bird.

### 2.5 Individual variation and selection pressures on reaction norms

Using all available data including one‐time breeders, we followed the one‐step approach demonstrated by Arnold et al. (2019), using bivariate generalized linear mixed models to assess individual‐variance and selection pressures on laying date—budburst date (LD‐BD) and clutch size—laying date (CS‐LD) reaction norms. We illustrate the approach here with the LD‐BD reaction norm. First, we constructed a mixed model for individual reaction norm properties:

$$\text{LD}\;∼\;\text{BD}+{\left(\text{LD}\right)}_{\mathrm{Year}}+{\left(\text{LD}\right)}_{\mathrm{ID}}+{\left(\text{LD}:\text{BD}\right)}_{\mathrm{ID}}$$

where LD is the laying date, BD is the fixed covariate of budburst date, ${\left(\text{LD}\right)}_{\mathrm{Year}}$ is the random effect of year on the intercept, ${\left(\text{LD}\right)}_{\mathrm{ID}}$ is the random effect of individual birds’ intercepts, and ${\left(\text{LD}:\text{BD}\right)}_{\mathrm{ID}}$ is the random effect of individual birds’ slopes. The individual random effect thus has the following variance–covariance structure:

$$P={\left[\begin{array}{cc} \sigma_{\mathrm{LD}}^{2} & \sigma_{LD,LD:BD}\\ \sigma_{LD,LD:BD} & \sigma_{LD:BD}^{2} \end{array}\right]}_{\mathrm{ID}}$$

where $\sigma_{\mathrm{LD}}^{2}$ is the among‐individual variance in intercepts, $\sigma_{LD:BD}^{2}$ is the among‐individual variance in slopes, and $\sigma_{LD,LD:BD}$ is the covariance between the random intercept and slope.

Next, we extended this model to a bivariate one by considering the equation

$$\omega =\mu_{\omega }+{\left(\omega \right)}_{\mathrm{ID}}$$

where ω is a vector of individual fitness values, $\mu_{\omega }$ is the mean fitness of the population, and ${\left(\omega \right)}_{\mathrm{ID}}$ is the deviation of individual birds’ fitness values from the mean. Since there is one fitness value for each individual, ${\left(\omega \right)}_{\mathrm{ID}}$ can be treated as a random effect of individual birds’ fitness in the bivariate model as a part of the residual variance, resulting in the following variance–covariance structure in the final model, with three levels at the individual effect:

$${\left[\begin{array}{ccc} \sigma_{\mathrm{LD}}^{2} & \sigma_{LD,LD:BD} & \sigma_{LD,\omega }\\ \sigma_{LD,LD:BD} & \sigma_{LD:BD}^{2} & \sigma_{LD:BD,\omega }\\ \sigma_{LD,\omega } & \sigma_{LD:BD,\omega } & \sigma_{\omega }^{2} \end{array}\right]}_{\mathrm{ID}}+\sigma_{\mathrm{year}}^{2}+\sigma_{\mathrm{residual}}^{2}$$

where $\sigma_{\omega }^{2}$ is the among‐individual variance in fitness, $\sigma_{LD,\omega }$ is the covariance between individual reaction norm intercept and fitness, $\sigma_{LD:BD,\omega }$ is the covariance between individual reaction norm slope and fitness, $\sigma_{\mathrm{year}}^{2}$ is the among‐year variance in laying date, and $\sigma_{\mathrm{residual}}^{2}$ is the residual variance in laying date. $\sigma_{LD,\omega }$ and $\sigma_{LD:BD,\omega }$ are thus selection differentials for individual intercepts and slopes, respectively, and represent the total selection on individual intercepts and slopes. If these selection differentials are concatenated into a vector S, then direct selection can further be obtained by calculating the selection gradients of individual intercepts and slopes β using

$$\beta =P^{‐1}S$$

where $P^{‐1}$ denotes the inverse matrix of P (Lande & Arnold, 1983).

Likewise, we assessed individual variation and selection on CS‐LD reaction norm using the same approach. We used lifetime breeding success (LBS), defined as the number of seven‐day‐old chicks a female produced throughout her reproductive lifespan, as the fitness measure (ω), as this measure presented the greatest sample size. In both models, we specified LBS as having a Poisson distribution. We discarded breeding data of the latest year (2019) under the assumption that all females breeding in that year had not yet completed their breeding careers (Nussey et al., 2005). We also removed second clutches and outlier observations with clutch sizes over 20 as they were likely the result of recording errors and/or of multiple birds breeding in the same nest box. In total, 1282 females, 3425 oak trees, 658 breeding observations for LD‐BD analyses, and 2107 observations for CS‐LD analyses were included in the final models. We used the R package *MCMCglmm* v2.26 (Hadfield, 2010), with 15 million iterations, 1.5 million burn‐ins, and a thinning interval of 1000 for each model. We used default priors for fixed effects, and inverse‐Wishart priors for both the random and residual effects, that is, V = diag(d), nu = d, where d is the number of dimensions of the VCV matrix. We confirmed model convergence by checking that autocorrelation <0.1, the trace plots did not show a time series (Hadfield, 2019), and the Heidelberger and Welch's convergence diagnostic was passed. Models reran with different priors for random and residual effects (V = diag(d), nu = 0.002; V = diag(d), nu = 1.002) showed the same conclusions (Appendix Tables A1, A2, A3, A4). We determined model posterior modes as significantly different from zero when their 95% credible intervals (CrI) did not overlap zero.

## 3 RESULTS

Of the 1447 blue tit females recorded in this study, 945 (65.3%) were one‐time breeders, and the maximum number of repeated breeding observations was seven (Appendix Table A5). The average LBS of the 1282 females used in the analyses was 8.47 chicks, with a range of 0 to 53, and a variance of 63.03 (Appendix Table A6, A7 and A6, A7).

### 3.1 LD‐BD relationship and plasticity

**FIGURE 3:**
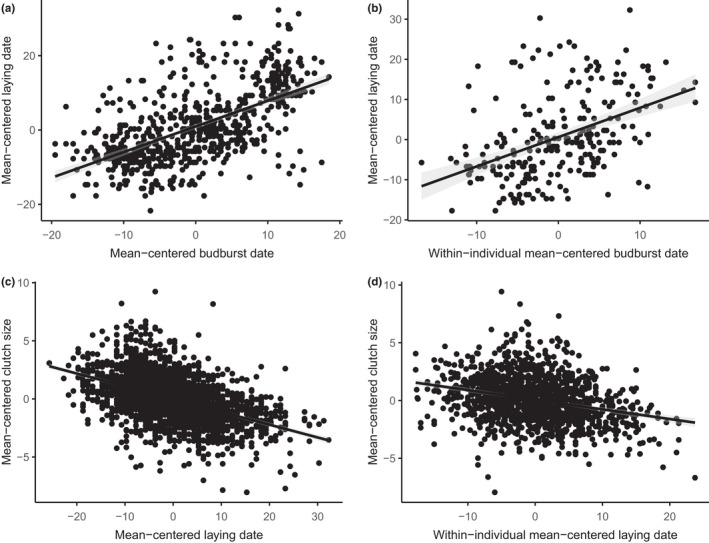
(a) Overall relationship and (b) within‐individual relationship between laying date and budburst date; and (c) overall relationship and (d) within‐individual relationship between clutch size and laying date. Shaded area represents 95% confidence interval. For (c) and (d), points are jittered about the y‐axis

The linear mixed model revealed a statistically significant and positive relationship between laying date and budburst date at the population level (Table 1, Figure 3A). Individuals account for roughly 3.3% of the total variance in laying date, while year accounts for 36.4%. We detected also in our linear model a statistically significant within‐individual effect of budburst date on laying date, with a positive slope of 0.730 ± 0.088 (*t*‐value = 8.30, variance explained by ID = 13.09, residual variance = 75.02), indicating the presence of an individual reaction norm (Figure 3B).

**TABLE 1:** The summary of the linear mixed model on overall laying date—budburst date relationship of Silwood Park blue tits and oaks, 2002–2019

Fixed effects			
	Estimate	*SE*	*t*‐value
Intercept	−0.10	1.63	−0.06
BD	0.16	0.06	2.59

Random effects		
Group	Variance	No. of groups
ID	2.59	517
Year	28.27	11
Residual	46.82	658

BD, budburst date.

### 3.2 Individual variation and selection on LD‐BD reaction norm

There was nonzero among individual variance in both reaction norm intercept and slope, and no covariance between the two properties. There was nonzero and negative covariance between individual intercepts and LBS, but that between the individual slopes and LBS did not differ from zero (Table 2). The selection gradient for individual intercepts was −0.51 (95% CrI: −0.86 to −0.20), and that of individual slopes was 0.255 (95% CrI: −0.43 to 1.08), indicating statistically significant directional selection on the intercept but not on the slope of the reaction norm.

**TABLE 2:** The model summary of laying date—budburst date (LD‐BD) reaction norm, showing variance‐covariance matrix for individual reaction norm intercept, slope and LBS, plus other random and fixed effects. Variances are on the diagonal while covariances are on the off‐diagonals

Variance‐covariance matrix			
	Post. Mean (95% CrI)		
	LD	LD:BD	LBS
LD	3.51 (0.64–7.13)^*^	0.10 (−0.18 to 0.40)	−1.41 (−2.30 to −0.52)^*^
LD:BD		0.16 (0.09 to 0.22)^*^	0.02 (−0.12 to 0.14)
LBS			1.39 (1.22 to 1.57)^*^

Random effects			
	Post. Mean	95% CrI	Effective sample size
year	11.98^*^	2.48 to 26.63	13,500
residual	46.96^*^	40.65 to 53.68	13,500

Fixed effects				
	Post. Mean	95% CrI	pMCMC	Effective sample size
Intercept	−0.88	−3.20 to 1.38	0.41	13,500
BD	0.18^*^	0.04 to 0.32	0.02	13,500

BD, budburst date; LBS, lifetime breeding success; LD, laying date.

* Statistically significant values.

### 3.3 CS‐LD relationship and plasticity

The linear mixed model revealed a statistically significant and negative correlation between clutch size and laying date (Table 3, Figure 3c). Individuals explained 32.6% of the total variance in clutch size, and year explained 16.4%. A statistically significant and negative within‐individual effect of laying date on clutch size was also found, with a slope of −0.08 ± 0.01 (*t*‐value = 14.64, variance explained by ID = 1.70, residual variance = 1.96), suggesting an individual CS‐LD reaction norm (Figure 3d).

**TABLE 3:** The summary of the linear mixed model on the clutch size—laying date relationship in the Silwood Park blue tits

Fixed effects			
	Estimate	*SE*	*t*‐value
Intercept	0.05	0.18	0.26
LD	−0.13^*^	0.00	−25.82

Random effects		
Group	Variance	No. of groups
ID	1.05	1447
year	0.53	17
residual	1.65	2278

Note

LD, laying date.

* Statistically significant values.

### 3.4 Individual variation and selection on CS‐LD reaction norm

There was among‐individual variance in CS‐LD intercepts and slopes, though variance in the slopes was smaller than that displayed in the LD‐BD model (Table 4). We detected a near‐zero but statistically significant and negative covariance in reaction norm intercept and slope, indicating that phenotypically a larger intercept is associated with a smaller slope. There was significant covariance between clutch size and LBS, and no covariance between individual slopes and LBS. Transforming total selection differentials resulted in a selection gradient of 0.39 (95% CrI: 0.24 to 0.53) on individual intercepts and a gradient of 0.42 (95% CrI: −0.52 to 1.18) on individual slopes, indicating directional selection pressure on individual intercepts for a larger clutch size, but not on plasticity.

**TABLE 4:** The model summary of the clutch size—laying date (CS‐LD) reaction norm, showing variance‐covariance matrix for individual reaction norm intercepts, slopes and LBS, and other random effects. Variances are on the diagonal while covariances are on the off‐diagonals

Variance‐covariance matrix			
	*Post. Mean (95% CrI)*		
	CS	CS:LD	LBS
CS	1.01 (0.79–1.22)^*^	−0.02 (−0.04–−0.00)^*^	0.42 (0.29–0.55)^*^
CS:LD		0.02 (0.02–0.02)^*^	0.00 (−0.02–0.02)
LBS			1.37 (1.20–1.54)^*^

Random effects			
	Post. Mean	95% CrI	Effective sample size
year	0.50^*^	0.19–0.92	13500
residual	1.44^*^	1.28–1.60	13500

Fixed effects				
	Post. Mean	95% CrI	pMCMC	Eff sample size
intercept	−0.05	−0.42–0.33	0.79	13500
LD	−0.14^*^	−0.15–−0.12	<0.001	13500

CS, clutch size; LD, laying date; LBS, lifetime breeding success.

* Statistically significant values.

## 4 DISCUSSION

### 4.1 Laying date—budburst date and clutch size—laying date relationships

We demonstrated a positive relationship between oak budburst and blue tit laying date, which was in line with some previous studies (e.g., Bourgault et al., 2010; Thomas et al., 2010), suggesting that blue tits used leafing as a cue for egg‐laying; but contradicted others (e.g., Nilsson & Källander, 2006; Schaper et al., 2011; Visser et al., 2002). Such discrepancy could be due to the spatial scale of tree phenology monitored. In this study, we used tree phenology data at a finer scale, and paired oak trees with nest boxes based on the foraging area of blue tits. Since tree budburst is tightly coupled with caterpillar emergence and abundance (Nilsson & Källander, 2006), our budburst data thus directly reflected the caterpillar availability to birds in the local environment, and as a result would be more likely to predict the timing of egg‐laying. This, coupled with the fact that individuals displayed varying laying dates with budburst, suggested oak budburst could be a cue for egg‐laying in this population.

Clutch size displayed a decline with laying date both at the population level and within the individual, consistent with the predicted outcome of the trade‐off between the two traits (Lack, 1954), and with previous studies (Brommer et al., 2003). An earlier laying date likely coincides better with the food abundance peak, and translates to heavier chicks with a higher chance of survival after fledging (Perrins, 1965). As spring passes, food availability diminishes and parents are unable to feed as many chicks as during the start of the season. Furthermore, feeding effort of parents does not increase proportionally with brood size (Gibb, 1955), and the larger the brood, the less food each chick receives. Considering these, it is therefore a better strategy to lay fewer eggs as the season progresses, so as to ensure success of all chicks in the brood. This ultimately creates the negative relationship between clutch size and laying date demonstrated in this study.

### 4.2 Among‐individual variation in laying date—budburst date and clutch size—laying date reaction norms

We demonstrated that individuals possess the ability to adjust laying date and clutch sizes under their own regimes, as there was significant within‐individual effects in both LD‐BD and CS‐LD relationships, as well as significant among‐individual variance in the slopes and intercepts of both reaction norms. Individual variance in laying date was smaller than that previous reported in this population (13.38 ± 3.65, *n* = 446, Thorley & Lord, 2015). Such difference could be attributed to the difference in sample size, or a result of heterogeneity in individual variance across years. While this heterogeneity could be of interest in itself (Cleasby et al., 2015), we did not specifically tested for this in the models in this study, so as to prevent overfitting (Ramakers et al., 2020).

Since trait variation is essential for natural selection, there is capacity for LD‐BD and CS‐LD reaction norms to be subjected to selection. This among‐individual variation could be attributed to two sources. First is a genetic system controlling the expression of reaction norms such that genetically related individuals display less variation than nonrelated individuals would. To detect genetic variation of the individual reaction norms, random regression animal models can be employed by fitting a genetic pedigree as a random effect. A second source of variation is the effect of the environment and circumstances. Birds have the ability to learn – those that have experienced a warmer spring begin egg‐laying earlier in the subsequent year and vice versa, for example (Nussey et al., 2005). Within a population, individuals may experience a unique set of environmental changes throughout their lifetimes based on their location, its associated microclimate, and through chance. Thus, each individual could develop varying reaction norm properties, optimized to their local environments (Brommer et al., 2003).

It is crucial as a next step to quantify the contributions of genes and the environment to the variation in reaction norm properties via heritability analyses. Heritability has been proven in laying date‐temperature reaction norms (Charmantier et al., 2008; Nussey et al., 2005), but not on LD‐BD and CS‐LD reaction norms, calling for further research effort. In addition, to examine environmental effects, it would be interesting to compare reaction norms of birds of different age and experienced mismatch. Grieco et al. (2002) experimentally demonstrated that blue tits were capable of learning from previous experience—birds that experienced a greater mismatch in the previous year would adjust their laying date to a greater degree in the subsequent year. If learning plays a critical role in shaping plasticity, one could expect individual variation in reaction norms to change with age and the degree of previous mismatch.

Furthermore, our results show that LD‐BD reaction norm slopes possess higher among‐individual variance than those of the CS‐LD reaction norm, both comparable because both were mean‐centered. Brommer et al. (2012) theorized that there should be an optimal reaction norm to maximize reproductive output in a particular set of environments, that is, in a particular population. When there is deviation of individual reaction norms from the optimum, fitness is reduced, and selection drives individuals toward the optimal reaction norm, ultimately decreasing among‐individual variance. The results here may thus mean that in our population, the clutch size–laying date reaction norm slope has already been pushed closer to the optimum by selection than has laying date–budburst reaction norm slope (Charmantier et al., 2008). Without heritability estimates, it is difficult to conclude that CS‐LD reaction norm had the capacity to evolve. Nevertheless, one could expect evolution on the CS‐LD reaction norm to halt before the LD‐BD reaction norm does, due to little and decreasing individual variation and diminishing selection strength in the former.

### 4.3 Selection on laying date—budburst date and clutch size—laying date reaction norms

Our results indicated directional selection pressure toward an earlier laying date and a larger clutch size, which is consistent with each other and with previous literature (Brommer et al., 2012; van Noordwijk et al., 1995; Thorley & Lord, 2015). An earlier laying date allows better synchrony with food abundance, and a larger associated clutch size, pushing individuals toward a higher overall reproductive output. Contrary to common findings (Brommer et al., 2005; Nussey et al., 2005), there was no significant covariance between the LD‐BD reaction norm intercept and slope, and only a small covariance in the CS‐LD reaction norm intercept and slope, meaning that in this population, selection on the intercept is unlikely to result in indirect selection on the slope.

The insignificant covariance between slope (plasticity) and fitness in both LD‐BD and CS‐LD reaction norms inferred that the more plastic females did not perform better or worse than the less plastic females in reproductive output. Selection gradients also indicated no directional selection on plasticity for both reaction norm slopes. In LD‐BD plasticity, this could be explained by the possibility that selection favors reaction norms that enable birds to achieve maximum synchrony with oak budburst. As the emergence of food peak is only momentary, there is a narrow window for birds to reproduce. This means that to ensure a well‐timed laying date with food abundance across years, an optimal reaction norm slope, that is, an intermediate response to budburst, is needed (Reed et al., 2006). When budburst phenology varies among years, as in our study (Appendix Table A3), an overly plastic female would hurry laying too much ahead of the caterpillar peak in an early‐budburst year, and delay laying too much in a late‐budburst year, thus falling out of synchrony. On the contrary, a nonplastic female would lay too late in an early‐budburst year, and too early in a late‐budburst year, and likewise fall out of synchrony. As such, the highest fitness should be associated with the optimal plasticity, and selection should drive individuals toward the single reaction norm slope in favor of more extreme ones. In other words, stabilizing selection would occur. In CS‐LD plasticity, the case is similar—an overly plastic female suffers a reduction in number of chicks produced greater than the gain from improved chick survival, and vice versa (Brommer et al., 2012). An essential next step, therefore, would be to examine stabilizing selection on reaction norm slope, which could be achieved by detecting directional selection on the square term of the slope (Brommer et al., 2012; Reed et al., 2006). However, as our current models did not allow this square term to be modeled (see below), we did not include stabilizing selection in this study.

The inability to detect directional selection in this study could also be attributed to limitations in estimating fitness. The Silwood blue tit population is open (Appendix Table A5), and LBS estimates are thus prone to errors, as females might have raised broods elsewhere, resulting in the underestimation of reproductive output overall, and an upward bias in LBS toward birds with more recorded breeding observations. Nevertheless, LBS remains one of the most widely used fitness measures (e.g., Brommer et al., 2005; Nussey et al., 2005; Slate et al., 2000) in wild populations. Finally, we did discover selection on the intercepts so it is likely that this measure is appropriate for our purpose.

### 4.4 Bivariate random regression models to estimate selection

In this study, we demonstrated a statistical approach capable of estimating among‐individual variance in reaction intercept and slope, covariance in intercept and slope, and selection differentials on intercept and slope simultaneously. This approach has advantages over a conventional two‐step method, which requires both the characterization of the among‐individual variance in reaction norm intercepts and slopes, and then calculating selection pressure by regressing a lifetime reproductive fitness measure on these intercepts and slopes (e.g., Nussey et al., 2005). In the latter, statistical errors would be carried over from one analysis to the next (Arnold et al., 2019). In addition, to perform the second step, one can either utilize estimates from a simple linear regression, or best linear unbiased predictors (BLUP) of random effects from mixed models in the first step (Brommer et al., 2012). The former allows only data of individuals with a fairly large number of repeated measurements, thus discarding potentially a large proportion of data, while the latter violates the assumption that BLUP values are derived when all variables affecting the response variable have been included (Brommer et al., 2012). Bivariate models do not have these limitations and are thus an advanced way to assess selection pressure. In addition, they allow also the estimation of selection on nonlinear reaction norms, by fitting a quadratic or higher order function as the individual trait–environment/trait–trait relationship (Arnold et al., 2019). These models are, however, unable to detect nonlinear selection pressures on plasticity, which requires the covariance between fitness and the square term of the slope. Since in the model the slope is developed from the within‐individual covariance between the focal trait and the environment/predictor trait, one cannot directly manipulate it to obtain a square term. As such, conventional methods will need to be employed for nonlinear selection analyses in the future.

## 5 CONCLUSIONS

Using breeding data of a wild blue tit population, along with tightly coupled oak phenology data, we examined whether laying date—budburst and clutch size—laying date reaction norms exist and have the potential to evolve. Laying date increased with budburst date, while clutch size decreased with laying date. We found both reaction norms to be present, with significant among‐individual variance in the properties of both, the intercept (individual laying date/clutch size) and the slope (plasticity). We found directional selection for an earlier laying date and a larger clutch size, but no directional selection of either laying date—budburst or clutch size—laying date plasticity. We suggest that stabilizing selection might be present instead. While research in phenotypic plasticity is gaining momentum, it will take further effort to unravel the mechanisms by which evolution of plasticity operates.

## CONFLICT OF INTEREST

The authors declare no conflicts of interest.

## AUTHOR CONTRIBUTIONS

**Heung Ying Janet Chik:** Conceptualization (equal); Data curation (equal); Formal analysis (equal); Investigation (equal); Resources (equal); Visualization (equal); Writing – original draft (equal). **Catalina Estrada:** Data curation (equal); Resources (equal); Writing – review & editing (equal). **Yiqing Wang:** Data curation (equal); Resources (equal); Writing – review & editing (equal). **Priyesha Tank:** Data curation (equal); Resources (equal); Writing – review & editing (equal). **Alex Lord:** Conceptualization (equal); Data curation (equal); Resources (equal); Writing – review & editing (equal). **Julia Schroeder:** Conceptualization (equal); Data curation (equal); Resources (equal); Supervision (equal); Writing – review & editing (equal).

## Data Availability

Data files and the R script used in our analyses are available at: https://doi.org/10.6084/m9.figshare.11856108.

## 1

**TABLE A1:** The model summary of LD‐BD reaction norm using default priors for fixed effects, and inverse‐gamma priors (V = diag(d), nu = 0.002) for random and residual effects. Number of iterations = 1 million, burn‐in = 100,000, thinning interval = 100

Variance‐covariance matrix			
	Post. Mean (95% CI)		
	LD	LD:BD	LBS
LD	2.53 (0.34–5.13)^*^	0.04 (−0.16 to 0.28)	−1.70 (−2.57–−0.78)^*^
LD:BD		0.01 (0.00 to 0.04)^*^	−0.00 (−0.13–0.12)
LBS			1.38 (1.21–1.56)

Random effects			
	Post. Mean	95% CI	Effective sample size
Year	14.68	2.72 to 32.35	9000
Residual	51.05	44.84 to 57.61	2378

Fixed effects				
	Post. Mean	95% CI	pMCMC	Eff sample size
Intercept	−0.95	−3.51 to 1.59	0.42	8915
BD	0.15^*^	0.01 to 0.28	0.03	7128

* Statistically significant values.

**TABLE A2:** The model summary of LD‐BD reaction norm using default priors for fixed effects, and inverse‐Wishart priors with V = diag(d) and nu = 1.002 for random and residual effects. Number of iterations = 1 million, burn‐in = 100,000, thinning interval = 100

Variance‐covariance matrix			
	Post. Mean (95% CI)		
	LD	LD:BD	LBS
LD	3.76 (0.62–7.75)^*^	0.11 (−0.16 to 0.46)	−1.58 (−2.44 to −0.62)^*^
LD:BD		0.10 (0.05 to 0.16)^*^	0.01 (−0.12 to 0.14)
LBS			1.39 (1.22 to 1.56)

Random effects			
	Post. Mean	95% CI	Effective sample size
Year	12.13^*^	2.51 to 26.54	9000
Residual	47.73^*^	41.21 to 54.35	5754

Fixed effects				
	Post. Mean	95% CI	pMCMC	Eff sample size
Intercept	−0.88	−3.10 to 1.48	0.42	9612
BD	0.17^*^	0.04 to 0.31	0.02	9738

* Statistically significant values.

**TABLE A3:** The model summary of CS‐LD reaction norm using default priors for fixed effects, and inverse‐gamma priors (V = diag(d), nu = 0.002) for random and residual effects. Number of iterations = 1 million, burn‐in = 100,000, thinning interval = 100

Variance‐covariance matrix			
	Post. Mean (95% CI)		
	CS	CS:LD	LBS
CS	1.18 (0.96–1.41)^*^	−0.02 (−0.03 to −0.00)^*^	0.41 (0.28 to 0.54)^*^
CS:LD		0.00 (0.00 to 0.00)^*^	−0.00 (−0.02 to 0.01)
LBS			1.37 (1.21 to 1.54)

Random effects			
	Post. Mean	95% CI	Effective sample size
Year	0.43^*^	0.15 to 0.83	9380
Residual	1.60^*^	1.43 to 1.77	7959

Fixed effects				
	Post. Mean	95% CI	pMCMC	Effective sample size
Intercept	−0.03	−0.36 to 0.34	0.86	9284
LD	−0.13^*^	−0.14 to −0.12	<0.001	8506

* Statistically significant values.

**TABLE A4:** The model summary of CS‐LD reaction norm using default priors for fixed effects, and inverse‐Wishart priors with V = diag(d) and nu = 1.002 for random and residual effects. Number of iterations = 1 million, burn‐in = 100,000, thinning interval = 100

Variance‐covariance matrix			
	Post. Mean (95% CI)		
	CS	CS:LD	LBS
CS	1.06 (0.84–1.28)^*^	−0.02 (−0.04 to −0.00)^*^	0.41 (0.28 to 0.55)^*^
CS:LD		0.01 (0.01 to 0.01)^*^	−0.00 (−0.02 to 0.02)
LBS			1.37 (1.21 to 1.54)

Random effects			
	Post. Mean	95% CI	Effective sample size
Year	0.50^*^	0.18 to 0.92	9000
Residual	1.47^*^	1.31 to 1.63	9000

Fixed effects				
	Post. Mean	95% CI	pMCMC	Effective sample size
Intercept	−0.04	−0.41 to 0.32	0.82	9000
BD	−0.14^*^	−0.15 to −0.12	<0.001	9000

* Statistically significant values.

**TABLE A5:** The number of female blue tits caught and associated number of repeated breeding observations, 2002–2019

Number of breeding observations	Number of females
1	945
2	282
3	143
4	55
5	14
6	6
7	2
Total	1447

**TABLE A6:** The summary of Silwood Park blue tit breeding data

Year	No. of nests	Mean LD (Range)	Var (LD)	Mean CS (Range)	Var (CS)	No. of 7‐ day‐old chicks	Mean no. of 7‐day‐old chicks per female
2002	103	12.60 (3 to 25)	28.65	9.70 (4–14)	3.33	854	8.29
2003	131	22.28 (5 to 34)	19.31	8.11 (1–13)	2.58	734	5.60
2004	153	23.14 (12 to 41)	26.91	8.86 (3–14)	3.19	991	6.48
2005	193	19.63 (6 to 34)	38.36	9.00 (1–15)	4.40	668	3.46
2006	82	24.56 (19 to 35)	12.27	9.27 (6–12)	1.90	676	8.24
2007	178	14.92 (7 to 28)	16.98	9.64 (4–19)	2.93	1248	7.01
2008	93	17.56 (5 to 30)	40.44	10.57 (6–16)	4.99	776	8.34
2009	122	13.75 (5 to 41)	34.09	10.98 (6–18)	4.16	811	6.65
2010	82	20.51 (7 to 42)	46.38	10.42 (5–14)	4.00	708	8.63
2011	134	13.47 (1 to 36)	23.23	10.01 (6–15)	2.31	1175	8.77
2012	170	15.67 (1 to 42)	64.18	9.22 (3–15)	5.69	483	2.84
2013	150	32.93 (24 to 51)	28.24	8.40 (3–17)	2.72	457	3.05
2015	134	21.61 (12 to 48)	57.23	8.66 (2–13)	3.32	529	3.95
2016	139	25.57 (10 to 49)	64.18	8.09 (4–13)	2.90	287	2.06
2017	135	12.68 (−3 to 42)	94.44	9.22 (4–14)	3.74	418	3.10
2018	108	21.17 (1 to 38)	22.10	9.58 (5–15)	2.99	490	4.54
2019	171	9.34 (−7 to 37)	70.66	9.55 (5–15)	3.20	1079	6.31

CS, clutch size; LD, laying date (0 = 1st April).

**TABLE A7:** The summary of Silwood Park oak budburst data. BD =budburst date (0 = 1st April), defined as when a tree reaches stage 2

Year	No. of oaks measured	Mean no. of oaks measured per nest box	Mean BD (range)	Var (BD)
2007	423	10.32	6.58 (1 to 19)	12.56
2008	661	7.18	18.39 (0 to 36)	70.30
2009	1032	8.82	12.31 (−6 to 36)	37.48
2010	1629	10.31	21.55 (6 to 51)	29.79
2011	1699	11.48	8.18 (−6 to 23)	13.09
2012	1813	11.62	10.60 (−10 to 49)	110.08
2013	1844	8.78	28.28 (18 to 52)	13.64
2015	346	2.98	18.48 (7 to 41)	35.00
2016	534	2.64	22.05 (12 to 36)	30.71
2017	477	2.59	13.91 (5 to 35)	37.48
2018	812	4.00	17.50 (8 to 30)	8.63
